# Lateralization of Motor Signs Affects Symptom Progression in Parkinson Disease

**DOI:** 10.3389/fneur.2021.711045

**Published:** 2021-07-27

**Authors:** Mazen Elkurd, Jijia Wang, Richard B. Dewey

**Affiliations:** ^1^Department of Neurology, UT Southwestern Medical Center, Dallas, TX, United States; ^2^Department of Applied Clinical Research, UT Southwestern Medical Center, Dallas, TX, United States

**Keywords:** Parkinson's disease, lateralization, asymmetry, progression, motor symptoms

## Abstract

**Background:** Asymmetry of motor signs is a cardinal feature of Parkinson disease which may impact phenotypic expression.

**Objective:** To investigate the relationship between lateralization of motor signs and symptom progression and severity during longitudinal observation for up to 4 years in a naturalistic study.

**Methods:** We analyzed data prospectively collected during the NINDS Parkinson Disease Biomarker Project (PDBP). We defined the Movement Disorder Society Revision of the Unified Parkinson Disease Rating Scale (MDS-UPDRS) part II as the primary measure of symptom progression. Left side predominant subjects were those whose lateralized motor scores on the MDS-UPDRS part III were ≥2 points higher on the left side than on the right side of the body. Multiple regression models (controlled for age, gender, education years, ethnicity, levodopa equivalent daily dose (LEDD) at baseline, and years with PD) were used to estimate the rate of symptom progression comparing left predominant (LPD) with non-left predominant (NLPD) subjects. A sensitivity analysis was performed using the same multiple regression models in the subgroups of low (0–26) or high (>27) MDS-UPDRS II score at baseline to determine if PD severity influenced the results.

**Results:** We included 390 participants, 177 LPD and 213 NLPD. We found that MDS-UPDRS part II progression from baseline to 48 months was faster in LPD compared to NLPD (0.6 points per year faster in LPD, *p* = 0.05). Additionally, the LPD group was statistically significantly worse at baseline and at 48 months in several subparts of the MDS-UPDRS and the Parkinson's Disease Questionnaire-39 (PDQ-39) mobility score. Significantly slower progression (difference of −0.8, *p* = 0.01) and lower score at 48 months (difference of −3.8, *p* = 0.003) was seen for NLPD vs. LPD in the group with lower baseline MDS-UPDRS part II score.

**Conclusion:** Left side lateralization was associated with faster symptom progression and worse outcomes in multiple clinical domains in our cohort. Clinicians should consider using motor predominance in their counseling regarding prognosis.

## Introduction

Parkinson disease is a progressive neurodegenerative condition of unknown etiology with no known cure. PD is a complex, heterogeneous condition which presents with various blends of motor and non-motor symptoms. These differing subtypes may vary considerably in rate of progression (1). This heterogeneity, combined with lack of any predictive tools, is an obstacle to accurate prognostic counseling of patients and complicates disease modifying research because different subtypes may respond differently to interventions. Identification of distinct phenotypic subtypes has proven an important area of research in the past decade (2), and numerous studies have been published to date examining the association of various motor and non-motor features with disease progression (3). Despite these efforts, significant uncertainty and controversy remains, and no tool exists for predicting the trajectory of any individual case (3, 4).

Asymmetry of motor signs is an important feature of PD which has been investigated as a potential predictor of disease severity or progression. Asymmetry is a cardinal feature of the human brain, with well-understood lateralization of functions including specialized areas for language and visuospatial processing (5). Asymmetry has also been described in pathological states including several neurodegenerative processes such as frontotemporal dementia (6), Alzheimer disease (7), and Huntington disease (8). In Parkinson disease, this asymmetry is important for diagnostic certainty and when absent poses a red flag against the diagnosis (9). Motor asymmetry in PD has been substantiated by imaging (10) and histopathologic examinations (11) demonstrating asymmetric nigrostriatal degeneration correlating with lateralized severity.

The clinical significance of asymmetry of motor symptoms in PD has not been extensively studied though there are reports linking lateralized symptoms to severity of motor and non-motor symptomatology. Amick et al. found that individuals with left-predominant symptoms exhibited poorer visual memory, whereas those with right-predominant features demonstrated poorer verbal memory (12). Dewey et al. found that right-sided onset of tremor appeared to be predictive of a lower risk of depressive symptoms (13). Cubo et al. evaluated 652 PD patients and noted that left-predominant disease was associated with worse motor and non-motor performance (14). A major limitation of this prior work was its cross-sectional nature. These provide evidence that motor asymmetry is associated with function at a given point in time. By contrast, we undertook to use the prospectively-collected dataset of the Parkinson Disease Biomarker Program (PDBP) to determine if motor laterality at baseline influenced future severity and progression during up to 4 years of follow-up. Based on prior work, our hypotheses were that (1) left-side predominant disease would be associated with worse motor and cognitive symptoms, and (2) that rate of progression of symptoms would be faster in those with left-side motor predominance.

## Methods

### Standard Protocol Approvals, Registrations, and Patient Consents

For this study, we analyzed data previously collected through the National Institute of Neurologic Disorders and Stroke (NINDS) PDBP. This was a prospective, multi-site longitudinal cohort study in which clinical data and biological samples were collected from participants with Parkinson disease as well as control subjects who were followed for up to 4 years. The methods of data collection have been previously published (15). The study protocol was reviewed and approved by the Institutional Review Board of the University of Texas Southwestern Medical Center, and at the other institutions where subjects were recruited and followed as part of this project. Written informed consent was obtained from all participants. The study was registered on clinicaltrials.gov with registration number NCT01767818. The study is reported in accordance with STROBE reporting criteria for cohort studies.

### Subjects

Subjects were recruited in this longitudinal cohort study at three sites in the United States: UT Southwestern Medical Center, Johns Hopkins University School of Medicine, and PennState Health Milton S. Hershey Medical Center between 2012 and 2017. Subjects had a diagnosis of idiopathic PD according to UK Brain Bank Criteria, were males or females aged 30 or older at the time of diagnosis, if untreated with dopaminergic agents had confirmation of dopamine transporter deficit by I-123 Ioflupane SPECT (DatScan), and if treated with dopaminergic agents had clinical evidence of a favorable response to treatment. Subjects suspected to have atypical parkinsonian disorders or secondary parkinsonian syndromes due to drugs, metabolic disorders or encephalitis were excluded from this analysis. Most subjects were initiated on anti-parkinsonian dopaminergic therapy at their physician's discretion prior to study entry, while a few entered the study unmedicated and were later placed on dopaminergic therapy. For this project, we included all available longitudinal data from baseline through 48 months, but due to a data anomaly in motor scoring introduced by a change of the clinical rater at one recruitment site after the month 12 assessment, we censored MDS-UPDRS part III data from 18 months onward from that site.

### Outcome Measures

We designated the primary outcome measure for this analysis to be the rate of change per year in the MDS-UPDRS part II score from baseline to month 48. Though the subparts and total score of the MDS-UPDRS have been found to increase in a linear fashion in longitudinal cohorts and serve as reliable measures of disease progression (16, 17), we chose the part II score as the primary outcome measure because it has been demonstrated to have a more robust association with PD disease duration independent of medication status compared to other UPDRS subscores, and thus may be a stronger predictor of progression in Parkinson disease (18). In addition, we were able to avoid censoring this variable at later visits at the site where the change of rater occurred because this is a patient-reported score. Secondary outcome variables were the rate of change from baseline to 48 months and average scores at 48 months of MDS-UPDRS I, III, IV and total scores (19), Montreal Cognitive Assessment (MoCA) (20), levodopa equivalent daily dose (LEDD) (21), Schwab & England Activities of Daily Living Scale (S&E) (22), a Global Composite Outcome (GCO) which combines part I-III of the MDS-UPDRS, S&E and MoCA (23), Epworth Sleepiness Scale (ESS) (24), University of Pennsylvania Smell Identification Test (UPSIT) (25), Hamilton Anxiety Scale (HAM-A) (26), Hamilton Depression Rating Scale (HAM-D) (27), and the Parkinson's Disease Questionnaire-39 (PDQ-39) scores for mobility, activities of daily living (ADL), emotional well-being, stigma, social support, cognition, communication, and bodily discomfort (28).

### Determination of Motor Lateralization

We used the MDS-UPDRS part III score at the baseline visit to determine lateralization. For each subject, we summed items 20–26 yielding a total lateralized motor score for each side of the body. Using a cut-off of ≥2 points according to the method described by Poletti et al. (29) participants were assigned to either the left predominant (LPD) or non-left predominant group (NLPD). The non-left predominant group consisted of those with right-predominant and symmetric motor scores.

### Statistical Analysis

Two-sample *t* test, Mann-Whitney *U* test, and chi-square tests were used to compare clinical features at baseline. We used univariate linear regression to estimate the subject-specific rate of change per year in each outcome measure and from this, predicted measurements at 48 months. In the same manner, we estimated the subject-specific difference between LPD and NLPD of MDS-UPDRS II at each visit. We then employed multiple regression models to identify whether there was a difference between left and non-left predominant subjects while controlling for demographic (age, gender, education, and ethnicity) and clinical (LEDD at baseline and PD duration) variables. A sensitivity analysis was performed using the same multiple regression models in the subgroups of low (0–26) or high (>27) MDS-UPDRS II score at baseline to determine if PD severity influenced the results. All statistical analyses were carried out using SAS 9.4 (SAS Institute Inc, Cary, NC). The graph was generated using Prism version 8 (GraphPad Software, LLC).

## Results

### Baseline Demographic and Clinical Characteristics

Our analysis included a total of 390 participants, of whom 177 were classified as left-predominant (LPD) and 213 were non-left predominant (NLPD). The baseline demographic and clinical characteristics of the participants are shown in Table 1. Age, years of education, gender, and ethnicity were similar in both groups. PD duration (calculated from the time of PD diagnosis) was longer in the LPD group (5.93 vs. 4.98 years, *p* = 0.015). MDS-UPDRS II, III, IV, and total scores were higher at baseline in the LPD group compared to NLPD. Participants in the LPD group also had significantly worse scores at baseline on the S&E (0.85 vs. 0.90, *p* = 0.016), ESS (8.14 vs. 7.04, *p* = 0.03), PDQ-39 mobility (16.7 vs. 11.5, *p* = 0.05), and GCO (0.12 vs. −0.12, *p* = 0.002).

**Table 1:** Baseline characteristics of PD subjects by groups (mean ± interquartile range).

**Variable**	**LPD**	**NLPD**	***P value***
Age	65 ± 14	64 ± 13	0.11
Education years	16 ± 5.0	16 ± 4.0	0.53
Gender (M)	62%	56%	0.25
Ethnicity (Hispanic or Latino)	6%	4%	0.39
Years with PD^*^	5.9 ± 6.0	5.0 ± 6.0	0.02
MDS-UPDRS part I	9.0 ± 8.0	8.1 ± 7.0	0.09
MDS-UPDRS part II^*^	10 ± 10	8.5 ± 9.0	0.02
MDS-UPDRS part III^*^	25 ± 17	19 ± 13	<0.001
MDS-UPDRS part IV^*^	2.8 ± 5.0	2.0 ± 3.0	0.04
MDS-UPDRS total^*^	47 ± 31	38 ± 28	<0.001
MoCA	26 ± 4.0	26 ± 3.0	0.39
S&E^*^	0.85 ± 0.10	0.90 ± 0.10	0.02
PDQ-39 Mobility^*^	17 ± 23	12 ± 15	0.05
PDQ-39 Activities of Daily Living	17 ± 21	16 ± 21	0.61
PDQ-39 Emotional Well-being	15 ± 21	13 ± 21	0.49
PDQ-39 Stigma	15 ± 25	13 ± 19	0.54
PDQ-39 Social Support	7.3 ± 8.3	5.6 ± 8.3	0.84
PDQ-39 Cognitive Impairment	19 ± 19	17 ± 19	0.22
PDQ-39 Communication	14 ± 25	13 ± 17	0.67
PDQ-39 Bodily Discomfort	24 ± 25	23 ± 25	0.81
GCO^*^	0.12 ± 0.80	−0.12 ± 0.68	0.002
HAM-A	6.5 ± 6.0	6.4 ± 7.0	0.69
HAM-D	5.1 ± 5.0	4.6 ± 4.0	0.22
Epworth^*^	8.1 ± 6.0	7.0 ± 6.0	0.03
UPSIT	19.5 ± 11.0	20.0 ± 12.0	0.48

*Years with PD is calculated from the time of diagnosis*.

* *Designates p ≤0.05*.

*MoCA, Montreal Cognitive Assessment; S&E, Schwab and England Activities of Daily Living Scale; GCO, global composite outcome; HAM-A, Hamilton Anxiety Scale; HAM-D, Hamilton Depression scale; Epworth, Epworth Sleepiness Scale; UPSIT, University of Pennsylvania Smell Identification Test*.

### Rate of Progression

**Figure 1:**
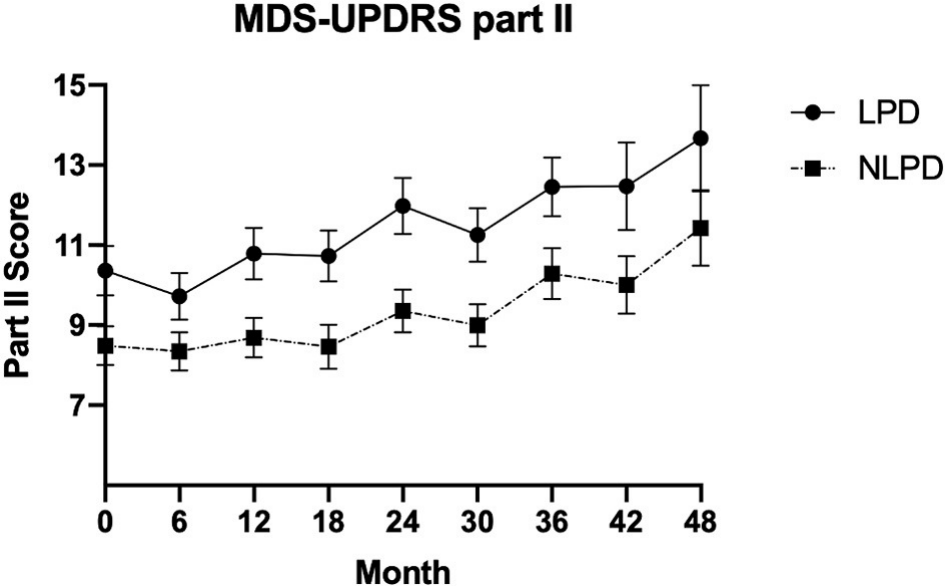
Mean value of MDS-UPDRS part II score in the two groups over time. Error bars represent standard error of the mean.

The primary outcome (rate of change in points per year of the MDS-UPDRS part II score) just met statistical significance showing that the LPD group progressed 0.6 points faster per year than the NLPD group (*p* = 0.05). A graph of the mean MDS-UPDRS part II score over time in the two groups is shown in Figure 1. We also performed exploratory analyses of all other outcome variables assessed and found no significant differences in the rate of progression between the groups in these other measures (Table 2).

**Table 2:** Multiple linear regression analysis showing the difference in progression rate (defined by rate of change per year) as measured by various clinical endpoints between NLPD and LPD subjects.

**Outcome**	**Estimate of NLPD–LPD**	***P value***
MDS-UPDRS part I	0.22	0.37
MDS-UPDRS part II^*^	−0.62	0.05
MDS-UPDRS part III	0.56	0.50
MDS-UPDRS part IV	−0.28	0.11
MDS-UPDRS total	−0.55	0.63
MoCA	0.13	0.35
LEDD	0.45	0.97
S&E	0.00	0.77
GCO	−0.03	0.36
Epworth	0.31	0.10
UPSIT	0.02	0.94
HAM-A	−0.26	0.34
HAM-D	0.04	0.83
PDQ-39 Mobility	−0.93	0.26
PDQ-39 Activities of Daily Living	−0.37	0.61
PDQ-39 Emotional Well-being	−0.17	0.83
PDQ-39 Stigma	−0.24	0.78
PDQ-39 Social Support	−0.26	0.70
PDQ-39 Cognitive Impairment	0.59	0.46
PDQ-39 Communication	−0.26	0.75
PDQ-39 Bodily Discomfort	−0.36	0.72

*Each model is adjusted for age, gender, education years, ethnicity, LEDD at baseline, and years with PD (as measured from time of diagnosis)*.

* *Designates p ≤ 0.05*.

*MoCA, Montreal Cognitive Assessment; S&E, Schwab and England Activities of Daily Living Scale; GCO, global composite outcome; HAM-A, Hamilton Anxiety Scale; HAM-D, Hamilton Depression scale; Epworth, Epworth Sleepiness Scale; UPSIT, University of Pennsylvania Smell Identification Test*.

### Symptom Severity at 48 Months

At 48 months, the average MDS-UPDRS II score in the LPD group was 3.4 points higher than the NLPD group (*p* = 0.01). By contrast, the MDS-UPDRS parts I, III and total scores were not significantly different. The MDS-UPDRS part IV was 1.4 points higher (*p* = 0.03) in the LPD group, and the PDQ-39 mobility score was 7.6 points higher in the LPD group (*p* = 0.03). The remaining measures did not show any significant differences in average scores at 48 months (Table 3).

**Table 3:** Multiple linear regression analysis showing difference in average measurement at 48 months between NLPD and LPD groups.

**Outcome**	**Estimate of NLPD–LPD**	***P value***
MDS-UPDRS part I	0.18	0.86
MDS-UPDRS part II^*^	−3.4	0.01
MDS-UPDRS part III	−2.7	0.41
MDS-UPDRS part IV^*^	−1.4	0.03
MDS-UPDRS total	−8.8	0.06
MoCA	0.45	0.44
LEDD	−15	0.78
S&E	0.03	0.16
GCO	−0.25	0.07
Epworth	0.68	0.36
UPSIT	−0.09	0.93
HAM-A	−1.4	0.20
HAM-D	−0.28	0.71
PDQ-39 Mobility^*^	−7.6	0.03
PDQ-39 Activities of Daily Living	−2.4	0.41
PDQ-39 Emotional Well-being	−2.1	0.46
PDQ-39 Stigma	−2.7	0.38
PDQ-39 Social Support	−3.1	0.19
PDQ-39 Cognitive Impairment	0.70	0.81
PDQ-39 Communication	−2.3	0.46
PDQ-39 Bodily Discomfort	−2.6	0.47

*Each model is adjusted for age, gender, education years, ethnicity, LEDD at baseline, and years with PD (as measured from time of diagnosis)*.

* *Designates p ≤ 0.05.*

*MoCA, Montreal Cognitive Assessment; S&E, Schwab and England Activities of Daily Living Scale; GCO, global composite outcome; HAM-A, Hamilton Anxiety Scale; HAM-D, Hamilton Depression scale; Epworth, Epworth Sleepiness Scale; UPSIT, University of Pennsylvania Smell Identification Test*.

### Sensitivity Analysis

We found significantly slower progression in the NLPD compared to LPD (difference of −0.8, *p* = 0.01) for the low MDS-UPDRS part II at baseline group, whereas there was no difference between NLPD and LPD in the high group (−2.7, *p* = 0.6). The average MDS-UPDRS II score at month 48 was significantly lower in NLPD in the low group (−3.8, *p* = 0.003) whereas no difference was seen between NLPD and LPD in the high group (−8.6, *p* = 0.6) (Table 4).

**Table 4:** Sensitivity analysis comparing rate of progression measured by change in MDS-UPDRS II score and average score at 48 months in LPD vs NLPD groups, stratified by baseline MDS-UPDRS II (high or low groups).

	**LPD vs. NLPD progression**	**LPD vs. NLPD score at 48 months**
Low baseline score	−0.7587 (*p* = 0.013^*^)	−3.7847 (*p* = 0.003^*^)
High baseline score	−2.6966 (*p* = 0.57)	−8.5878 (*p* = 0.599)

* *Designates p ≤ 0.05*.

## Discussion

In this analysis of a large multi-center longitudinal cohort of PD subjects, we demonstrated that individuals with left-predominant PD motor symptoms exhibit more rapid symptom progression over a 48 month period as measured by MDS-UPDRS part II scores. Our data also confirmed previously reported observations that those with left-predominant disease have a higher disease burden when compared to those with opposite laterality or symmetric motor signs. Our finding that both symptom scores and PD-related quality of life are worse in LPD subjects highlights the importance of this finding for counseling patients. Our results also add to the sparse literature regarding the durability of these effects showing persistent differences at 48 months in many of the same measures that differed at baseline. An additional interesting finding was that the LPD group had the diagnosis of PD for ~1 year longer than those without left-predominant disease. This observation may be related to their higher disease burden, which could ostensibly lead individuals to seek medical attention and thus arrive at a diagnosis earlier than their non-left dominant counterparts. The longer disease duration does not, however, explain the increased disease burden or faster progression of MDS-UPDRS part II scores because we controlled for disease duration in our analysis.

Our sensitivity analysis validated the direction of the main findings but revealed that this difference between NLPD and LPD progression and severity at 48 months held up only for the group with milder symptoms at baseline. While our study does not permit drawing firm conclusions about why this was so, we speculate that because more advanced cases (i.e., those with high baseline MDS-UPDRS II scores), have more extensive Lewy body pathology on both sides of the brain, the clinical differences between NLPD and LPD are therefore no longer present.

Our study had several limitations, most importantly that handedness was not documented in the original data set which prevented us from grouping subjects into dominant vs. non-dominant sides of predominance. However, since 90% of the population is right-handed (30), this likely did not affect the results. Other limitations included our need to censor MDS-UPDRS part III data from one site, variable lengths of follow-up of participants requiring us to model the subject-specific rates of progression using statistical techniques, and the lack of a universally agreed-upon method for determining asymmetry in PD. For instance, the method we used lacks sensitivity to detect asymmetry in very mild cases due to the ceiling effect, while alternative methods such as the UPDRS asymmetry index (14, 31) lack discriminant sensitivity at the high end of the MDS-UPDRS scale when subjects have more severe disease.

Though our study supports previous work showing a higher disease burden in LPD patients, and adds the finding of increased progression rate in MDS-UPDRS part II score, it remains unclear why LPD patients do worse. One potential biological explanation may be the existence of an increased dopaminergic reserve or more efficient motor network in the dominant left hemisphere of the 90 percent of individuals who are right-handed (30), thereby delaying the manifestations of motor symptoms on the contralateral side of the body (32). Another possible explanation is that more strenuous exercise and frequency of use of the dominant side of the body may be neuroprotective of dopamine networks thus accounting for the higher disease burden on the non-dominant side. This theory was bolstered by an experiment conducted in a rat model of PD created by unilateral injection of 6-hydroxydopamine into the striatum resulting in contralateral limb dysfunction and preferential use of the good limb. Investigators noted that when the healthy limb was immobilized forcing the rat to use the impaired limb, the expected contralateral limb dysfunction was prevented suggesting that frequency of use of the affected limb plays a significant role in motor dysfunction related to neurodegeneration (33).

The more rapid symptom progression and greater motor and non-motor symptom severity at baseline and 48 months are important clinical considerations. This information can be used for counseling patients regarding prognosis in a disease with notorious heterogeneity. Beyond this, the ability to determine which patients may progress at a more rapid rate in the future could prove invaluable in the elusive quest for disease-modifying therapy for PD where many therapeutics have been tested and failed. A major obstacle to demonstrating disease-modifying activity of a drug is the variable rate of symptom progression in study subjects which thus requires either a very large number of subjects or a long period of study or both. By recruiting study participants who are likely to have a rapid rate of progression, investigators may be able to more quickly identify the signal of disease-modifying activity. While lateralization of motor signs is not likely to be a sufficiently powerful predictor on its own for identification of fast progressors, it may wellserve as one of several selection criteria which could improve future clinical trial designs.

## Conclusion

Our study provides evidence from a longitudinally-followed cohort of PD subjects that left-predominant motor symptoms are associated with more rapid progression of symptoms and a higher disease burden at baseline and 48 months. These findings provide valuable insight into the potential disease course of an individual patient and may prove helpful for patient counseling and for recruiting likely fast progressors into clinical trials of disease modifying agents. Further prospective studies in independent cohorts are needed to confirm and extend these observations.

## Data Availability Statement

Publicly available datasets were analyzed in this study. This data can be found here: https://pdbp.ninds.nih.gov/parkinsons-data.

## Ethics Statement

The studies involving human participants were reviewed and approved by University of Texas Southwestern Medical Center IRB and by the IRBs at the other institutions who recruited subjects for this study. The patients/participants provided their written informed consent to participate in this study.

## Author Contributions

ME: study conception and draft manuscript preparation. ME and RD: study design, analysis and interpretation of results, and manuscript editing. JW: data analysis and statistical computation and statistical methods section writing. All authors reviewed the results and approved the final version of the manuscript.

## Conflict of Interest

RD reports personal fees (consulting) from Amneal, Acorda, Supernus, Teva, Adamas, US WorldMeds, Acadia, and Lundbeck, outside the submitted work. The remaining authors declare that the research was conducted in the absence of any commercial or financial relationships that could be construed as a potential conflict of interest.

## Publisher's Note

All claims expressed in this article are solely those of the authors and do not necessarily represent those of their affiliated organizations, or those of the publisher, the editors and the reviewers. Any product that may be evaluated in this article, or claim that may be made by its manufacturer, is not guaranteed or endorsed by the publisher.
